# Systemic treatment of patients with metachronous peritoneal carcinomatosis of colorectal origin

**DOI:** 10.1038/srep18632

**Published:** 2015-12-21

**Authors:** T. R. van Oudheusden, L. G. Razenberg, Y. R. van Gestel, G. J. Creemers, V. E. Lemmens, I. H. de Hingh

**Affiliations:** 1 Department of Surgery, Catharina Hospital, Michelangelolaan 2, 5623 EJ Eindhoven, the Netherlands; 2 Department of research, Netherlands Comprehensive Cancer Organization (IKNL), postbus 213, 5600 AE Eindhoven, The Netherlands; 3 Department of Oncology, Catharina Hospital, Michelangelolaan 2, 5623 EJ Eindhoven, the Netherlands; 4 Department of Public Health, Erasmus MC University Medical Centre, Rotterdam, The Netherlands

## Abstract

Combining chemotherapy and targeted therapies has resulted in an enhanced survival in metastatic colorectal cancer (mCRC) patients. However, the result of this palliative treatment in patients with metachronous peritoneal carcinomatosis (PC) remains unknown. The current population-based study aims to investigate the use and effect of palliative systemic treatment in patients with metachronous PC of colorectal origin. Data on metachronous PC were collected between 2010 and 2011 for all patients who were diagnosed with M0 colorectal cancer between 2003 and 2008 in the Dutch Eindhoven Cancer Registry. Patient demographics and detailed data on chemotherapeutic treatment were collected and compared. Ninety-two patients with metachronous PC received chemotherapy in a palliative setting compared to 94 patients without treatment. In 36 patients, Bevacizumab was added to the treatment (39%). Overall survival was 3.4, 13, and 20.3 months in the no treatment, systemic treatment and systemic treatment + Bevacizumab respectively (P < 0.001). Male gender was a positive predictor and right sided primary tumor location a negative predictor of receiving bevacizumab. Approximately 40% of patients with metachronous PC received bevacizumab in addition to chemotherapy. Treatment with systemic chemotherapy in combination with bevacizumab may increase survival in a patients with metachronous colorectal PC.

Overall survival for patients with colorectal peritoneal carcinomatosis (PC) is dismal and typically averages around 6 months^1^. After the implementation of cytoreductive surgery and hyperthermic intraperitoneal chemotherapy (HIPEC), 5-year survival rates of up to 40% have been reported while some patients even appear to be cured^2^,^3^,^4^. Unfortunately, the majority of PC patients do not qualify for this aggressive treatment due to the presence of other distant metastases, a poor condition, advanced disease or other reasons. In these patients systemic treatment with palliative intent is used in increasing frequency^5^.

Improvements in the systemic treatment of patients with metastatic colorectal cancer (mCRC) have resulted in extended survival. Currently, combining chemotherapy and targeted therapy is considered to be a standard of care for these patients^6^,^7^. However, no data from randomised controlled trials are available that have specifically addressed systemic treatment in PC patients. The sparse data available are derived from small subgroup analyses and population-based data^8^,^9^,^10^. Without exception, these studies show improved survival and support a role for modern combination therapy. These studies concern patients with synchronous PC where the primary tumor and PC were diagnosed simultaneously. However, in at least half of the patients, PC is diagnosed metachronously, after initial curative treatment for colorectal cancer^11^. So far, no data are available on the use and effect of palliative modern chemotherapy with or without targeted therapies in the latter group of patients. Therefore, the aim of the current population-based study is to investigate the use and effect of palliative systemic treatment in patients with metachronous PC of colorectal origin.

## Methods

### Patient selection

Data concerning patients presenting with metachronous PC of colorectal origin were extracted from the Eindhoven Cancer Registry that collects all data of patients with newly diagnosed cancer in the Southern part of the Netherlands^10^. This area comprises approximately 2.4 million inhabitants and has 10 community hospitals, 6 pathology departments and 2 radiotherapy institutions. All data are registered by trained registry managers who prospectively collect patient and tumor characteristics from medical charts.

Data on metachronous metastases were additionally collected between 2010 and 2011 for all patients who were diagnosed with M0 colorectal cancer between 2003 and 2008 in the Dutch Eindhoven Cancer Registry. Survival data were available until January 2014. Metachronous PC was defined as an interval of at least 3 months between primary tumor and PC diagnosis. Patient demographics and details concerning chemotherapeutic treatment and Bevacizumab (being the standard choice for targeted therapies at time of the current study) were collected and compared. Patients that underwent curative surgery for PC (CRS + HIPEC) were excluded from this study. Patients receiving targeted therapy prior to PC diagnosis and those who did not undergo a curative primary tumor resection were excluded from the analyses. Treatment and decisions were performed in accordance with national guidelines and regulations. The study protocol was approved and carried out in accordance with the Medical research Ethics Committees United (MEC-U).

### Statistical analysis

Patient and tumor characteristics were compared between patients with or without systemic treatment using the Chi square test. Next, the percentages of patients who additionally received Bevacizumab were calculated among those that received systemic treatment. Univariable and multivariable logistic regression analysis were used to identify predictors of treatment with Bevacizumab. Only variables with P < 0.10 in the univariate analysis were included in the multivariable analysis. The predictors were depicted as odds ratios with their 95% confidence intervals. The effect of systemic treatment on mortality was investigated using multivariable cox regression analyses and depicted as hazard ratios. Survival was determined using the Kaplan-Meier method and compared using a Log-rank test. Survival was defined as time from diagnosis of PC to death or end of follow up period (January 2014). All tests were two sided and p-value < 0.05 was considered to be significant. Statistical analyses were performed using SAS/STAT statistical software (SAS system 9.3, SAS Institute, Cary, North Carolina, USA).

## Results

**Figure 1:**
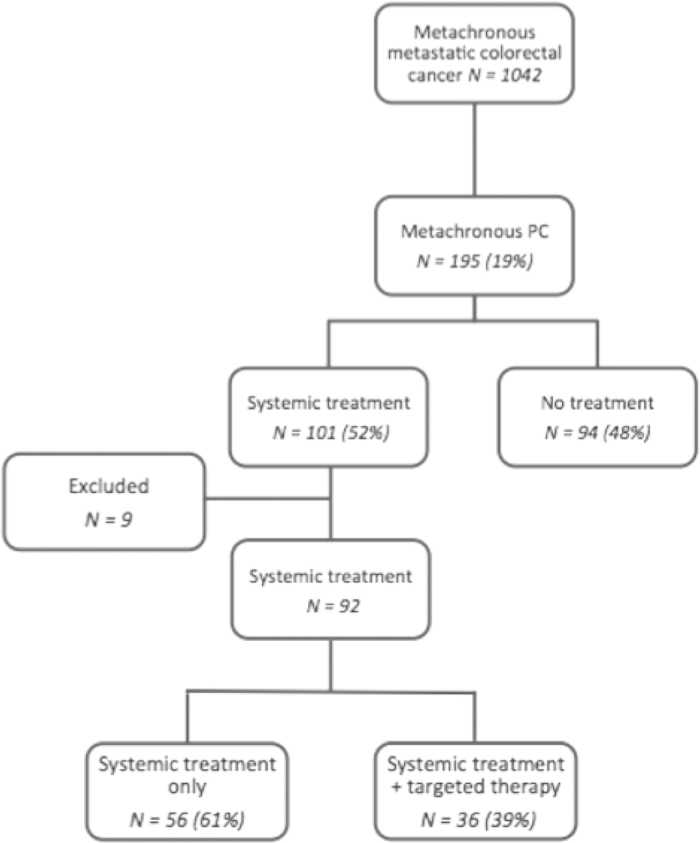
Study population.

Altogether, 1042 patients with primary colorectal cancer diagnosed between 2003 and 2008 developed metachronous metastases up until 2011. From these patients, 195 (19%) were diagnosed with metachronous PC and 101 among them (52%) received systemic treatment in a palliative setting. Nine of the latter patients were excluded from further analysis since they received targeted therapy prior to diagnosis of PC. Of the remaining patients, 36/92 patients (39%) received systemic treatment including Bevacizumab (Fig. 1).

### Treatment & patient characteristics

Patient and tumor characteristics were compared between patients with or without systemic treatment (Table 1). Patients receiving systemic treatment were younger than those receiving no treatment (67% vs. 31% with age <70, P < 0.01). Also, they displayed less comorbidities (48% vs. 68%, P < 0.01) and were more often diagnosed with other distant metastases (65% vs. 50%, P = 0.04). Gender, tumor differentiation, location, histology or T and N-stage did not differ between the two groups.

Table 2 shows the percentages of patient and tumor characteristics for those receiving additional Bevacizumab treatment among the total group of patients receiving systemic treatment. The multivariable odds ratios (OR’s) are provided for variables with a P-value < 0.10 in the univariate analysis. Among males, 51% received Bevacizumab compared to 26% among females (P = 0.01). In the age category below 70, 48% received Bevacizumab compared to 20% above 70 (P = 0.01). The group of patients without comorbidities received Bevacizumab more often (42% vs. 30%, P = 0.07). Regarding the primary tumor location, patients with left, right, rectum/rectosigmoid and overlapping/NOS as primary location received Bevacizumab in 60, 42, 30 and 33% of cases (P = 0.01).

The remaining factors tumor differentiation, histology, T-stage, N-stage and M-stage were not significantly different in the groups. Calculation of odds ratio’s showed male gender to be a positive predictor (OR 2.68, 95% CI 1.0–7.4) and right sided primary tumor location (OR 0.20, 95% CI 0.1–0.6) to be a negative predictor for treatment with Bevacizumab.

### Survival

**Figure 2:**
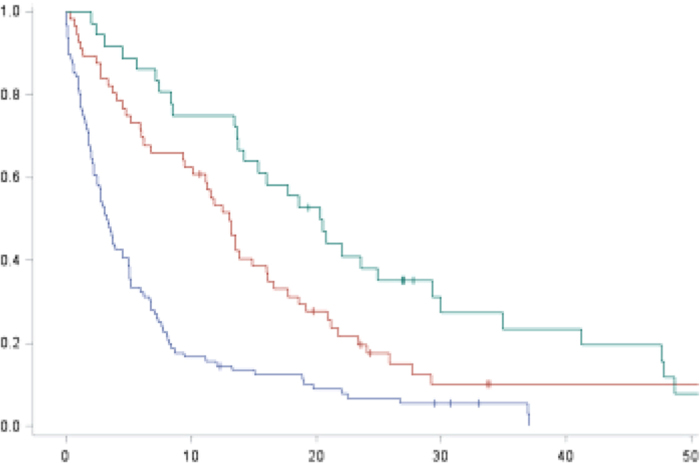
Kaplan Meier of survival of patients without systemic treatment (blue), with systemic treatment (red) or systemic treatment and bevacizumab (green).

Median overall survival of the entire PC group was 7.2 months (95% CI 5.2–9.4 months). Median overall survival was only 3.4 months (95% CI 2.5–4.9 months) in patients who did not receive systemic treatment, 13.0 months (95% CI 9.5–16.0 months) for patients who received systemic chemotherapy only and 20.3 months (95% CI CI13.7–29.3 months) for patients treated with chemotherapy and Bevacizumab (P < 0.001) (Fig. 2). Next, survival of patients receiving chemotherapy was compared to patients receiving both chemotherapy and Bevacizumab with median survival being significantly longer in the group receiving both treatments (P = 0.04).

Predictors of death for patients with metachronous PC are shown in Table 3 and presented as hazard ratios (HR). Age above 70 was associated with an increased risk of death (HR 1.86, 95% CI 1.29–2.68). The administration of both chemotherapy (HR 0.51, 95% CI 0.35–0.73) and chemotherapy with bevacizumab (HR 0.35, 95% CI 0.22–0.56) was associated with a decreased risk of death. When comparing chemotherapy only to chemotherapy and Bevacizumab, the hazard ratio was 0.69 (95% CI 0.42–1.12).

## Discussion

The data reported in this study suggest that palliative chemotherapy provided to patients diagnosed with metachronous peritoneal carcinomatosis of colorectal origin improved survival. Furthermore, the addition of Bevacizumab might improve survival even further.

While a positive effect of adding targeted therapy to chemotherapy has previously been established for synchronous PC^10^, it remained unknown if such treatment would also benefit patients in the metachronous setting. These patients differ from the synchronous group since they have already undergone surgery for the primary tumor with curative intent and developed the metastases during follow up. Moreover, it has been shown that survival after metachronous metastases of colorectal origin is different compared to the synchronous setting with better results in the metachronous subgroup of patients^12^. This difference might be attributable to a more aggressive tumor biology in those patients presenting with synchronous metastases^13^. The current data showed that the addition of Bevacizumab to chemotherapy resulted in a median survival of 20 months compared to 13 months for patients treated with chemotherapy only. Bevacizumab was the standard choice of treatment for metastasised colorectal cancer during the course of this study and therefore the observed effect may be specifically attributed, at least in part, to this biological agent. However, several other factors which probably play a role in patient eligibility for the various palliative treatment options may affect outcome as well.

Metachronous PC is diagnosed in 4% of patients with colorectal carcinoma and is know to result in a median survival of only a few months^14^,^15^,^16^. The introduction of cytoreductive surgery and HIPEC during the last two decades has, in selected groups, resulted in a median survival of up to 60 months after diagnosis^17^,^18^. Unfortunately, the majority of patients cannot benefit from this particular treatment since patients have to be fit for surgery with limited peritoneal disease and an absence of distant metastases^19^. Therefore, additional palliative treatment options must be investigated for these patients.

The use of targeted monoclonal antibodies has improved overall and progression free survival in mCRC^20^,^21^. However, these improvements have been mainly demonstrated in patients with metastases to the liver or lung. These organs are well vascularised making intravenous chemotherapy readily available at these metastatic sites as opposed to peritoneal deposits, which are located in a more isolated part of the human body. Klaver *et al.* demonstrated this phenomenon by analysing two trials that included mCRC patients in the Netherlands^9^: the survival benefit of systemic chemotherapy with or without targeted therapies is significantly less in PC than in non-PC metastasized patients. A similar analysis by Franko *et al.* led to similar conclusions^8^. However, these trials have in common that a relatively small percentage of metastasized patients had PC.

Recently, results of treatment with chemotherapy and targeted therapies have also emerged specifically for PC. Zani *et al.* compared a PC group treated with modern chemotherapy including targeted therapies to a group treated with outdated regimens and showed an increased median survival of 16.3 vs. 8.9 months in the former^22^. Another recent study by Chua *et al.* described a cohort of 294 patients with PC in a palliative setting. Combination chemotherapy regimens (5-FU based with oxaliplatin or irinotecan) resulted in a median survival rate of 15 months compared to 11 months using single agent 5FU regimens^23^. Interestingly, median survival appeared to be further increased by adding targeted therapies such as bevacizumab, cetuximab or panitumumab, resulting in a median survival of 23 months. Both studies included a mixed population of metachronous and synchronous presentation of PC. In addition, similar findings have been reported in a synchronous PC only cohort, with an increased median survival of 10.1 to 18.2 months after the addition of targeted therapies^10^.

Certain biases could not be evaded in the evaluation of the present cohort leading to a suggestion of increased survival when targeted therapies were added to palliative chemotherapy. The majority of patients receiving targeted therapies had less comorbidities and were younger, both conditions favouring enhanced survival. Nonetheless, in terms of hazard ratios, combining targeted therapies with chemotherapy proved to be a protective factor in multivariable analysis. However, when leaving out the patients who received no treatment, the addition of Bevacizumab did not significantly decrease the chance of death probably due to small sample size. Therefore the role of Bevacizumab in the treatment of metachronous PC from colorectal origin should be regarded as promising but needs further clinical investigation.

In addition, it stands to reason that considerations taken into account while deciding whether or not, to what extent and what type of chemotherapeutics and targeted therapies will be administered are patient and caretaker dependent^24^. Moreover, a significant proportion of patients had also other distant metastases. It is therefore uncertain to what extent increased survival can be attributed to the treatment of the peritoneal deposits in these patients, especially so since the effectiveness of targeted therapies in non-peritoneal metastases is supported by stronger evidence. Still, since many patients receive palliative treatment because of the presence of distant metastases they are a common phenomenon and therefore reflect clinical daily practice. Lastly, the extent of disease is impossible to assess since diagnostic modalities lack the ability to adequately estimate severance of disease. However, all patients were in a palliative setting, which means that either the patient condition wouldn’t allow surgery, or the presence of either extra-abdominal metastases or severe intra-abdominal metastases made surgery contra-indicated.

To our knowledge, no studies have reported on the added value of Bevacizumab to palliative chemotherapy in patients with metachronous PC. The results of the current study suggest that treatment with palliative systemic chemotherapy especially in combination with Bevacizumab may significantly improve survival in these patients. However, due to the nature of this population-based study potential selection bias is inevitably and the results should therefore be interpreted cautiously.

## Additional Information

**How to cite this article**: van Oudheusden, T. R. *et al.* Systemic treatment of patients with metachronous peritoneal carcinomatosis of colorectal origin. *Sci. Rep.* **5**, 18632; doi: 10.1038/srep18632 (2015).

## Figures and Tables

**Table 1:** Patient and tumor characteristics in patients treated with and without systemic chemotherapy.

Variable	No systemic treatment (%)	Systemic treatment (%)	P-value
	N = 94	N = 92	
Gender			
Female	54 (57.4)	43 (46.7)	0.14
Male	40 (42.6)	49 (53.3)	
Age (years)			
<70	29 (30.9)	62 (67.4)	**<0.01**
>70	65 (69.1)	30 (32.6)	
Comorbidity			
No	30 (31.9)	48 (52.2)	**<0.01**
Yes	64 (68.1)	44 (47.8)	
Tumor differentiation			
Good	4 (4.3)	5 (5.4)	0.95
Moderate	53 (56.4)	52 (56.5)	
Poor/undifferentiated	23 (24.5)	20 (21.7)	
Unknown	14 (14.9)	15 (16.3)	
Primary Location			
Left	32 (34.0)	41 (44.6)	0.10
Right	46 (48.9)	37 (40.2)	
Rectum/rectosigmoid	15 (16.0)	9 (9.8)	
Overlapping/NOS	1 (1.1)	5 (5.4)	
Histology			
Mucinous	21 (22,3)	26 (28.3)	0.62
Adenocarcinoma	70 (74.5)	64 (69.6)	
Signet ring cell	2 (2.1)	2 (2.2)	
Unknown	1 (1.1)	0	
T-stage			
T1,T2	6 (6.4)	3 (3.3)	0.57
T3	65 (69.1)	68 (73.9)	
T4	23 (24.5)	21 (22.8)	
N-stage			
N0	29 (30.9)	36 (39.1)	0.15
N1	31 (33.0)	35 (38.0)	
N2	32 (34.0)	21 (22.8)	
NX	2 (2.1)	0	
M-status			
PC only	47 (50.0)	32 (34.8)	**0.04**
PC + distant	47 (50.0)	60 (65.2)	

**Table 2:** Percentages and multivariable predictors of patients treated with Bevacizumab among those treated with systemic chemotherapy.

Variable	Systemic treatment	Bevacizumab (%)	Chi Square P-value	OR (95% CI)
Gender				
Female	43	11 (25.6)	**0.01**	**ref**
Male	49	25 (51.0)		**2.68 (1.0–7.4)**
Age (years)				
<70	62	30 (48.4)	**0.01**	**ref**
>70	30	6 (20)		0.37 (0.1–1.2)
Comorbidity				
No	48	23 (41.8)	**0.07**	**ref**
Yes	44	13 (29.5)		0.39 (0.1–1.2)
Tumor differentiation				
Good	5	3 (60.0)	0.56	
Moderate	52	22 (42.3)		
Poor/undifferentiated	20	6 (30.0)		
Unknown	15	5 (33.3)		
Primary Location				
Left	41	21 (51.2)	**0.01**	**ref**
Right	37	7 (18.9)		**0.20 (0.1—0.6)**
Rectum/rectosigmoid	9	5 (55.6)		0.72 (0.2–3.5)
Overlapping/NOS	5	3 (60.0)		0.90 (0.1–6.7)
Histology				
Mucinous	26	7 (19.4)	0.32	
Adenocarcinoma	64	28 (43.8)		
Signet ring cell	2	1 (50.0)		
	0			
T-stage				
T1,T2	3	1 (33.3)	0.66	
T3	68	25 (36.8)		
T4	21	10 (47.6)		
N-stage				
N0	36	14 (38.9)	0.78	
N1	35	15 (42.9)		
N2	21	7 (33.3)		
M-status				
PC only	32	15 (68.2)	0.27	
PC + distant	60	21 (35.0)		

OR: odds ratio. CI; confidence interval.

*The included variables were determined based on univariate logistic regression variables with P < 0.10.

**Table 3:** Predictors of death among colorectal cancer patients with metachronous PC (COX-regression analyses).

Variable	Dead (%)	P-value	HR (95% CI)
Gender			
Male	91.0	0.86	ref
Female	91.8		1.13 (0.82–1.56)
Age (years)			
<70	86.8	**0.03**	ref
>70	95.8		**1.86 (1.29–2.68)**
Comorbidity			
No	88.5	0.22	ref
Yes	93.5		1.18 (0.85–1.56)
Primary tumor location			
Left	90.4	0.76	ref
Right	91.6		0.97 (0.68–1.37)
Rectum/recto sigmoid	95.8		**1.73 (1.05–2.85)**
Overlapping/NOS	83.3		1.76 (0.68–4.55)
Systemic therapy			
No systemic therapy	95.7	0.10	ref
Chemotherapy only	87.5		**0.51 (0.35–0.73)**
Chemotherapy + Bevacizumab	86.1		**0.35 (0.22–0.56)**

HR: odds ratio. CI; confidence interval.

## References

[b1] Sadeghi B. *et al.* Peritoneal carcinomatosis from non-gynecologic malignancies: results of the EVOCAPE 1 multicentric prospective study. Cancer 88, 358–363 (2000). 10640968 10.1002/(sici)1097-0142(20000115)88:2<358::aid-cncr16>3.0.co;2-o

[b2] Elias D., Quenet F. & Goere D. Current status and future directions in the treatment of peritoneal dissemination from colorectal carcinoma. Surg Oncol Clin N Am 21, 611–623 (2012). 23021719 10.1016/j.soc.2012.07.014

[b3] Sugarbaker P. H. & Ryan D. P. Cytoreductive surgery plus hyperthermic perioperative chemotherapy to treat peritoneal metastases from colorectal cancer: standard of care or an experimental approach? Lancet Oncol 13, e362–369 (2012). 22846841 10.1016/S1470-2045(12)70210-3

[b4] Goere D. *et al.* Is there a possibility of a cure in patients with colorectal peritoneal carcinomatosis amenable to complete cytoreductive surgery and intraperitoneal chemotherapy? Ann surg 257, 1065–1071 (2013). 23299520 10.1097/SLA.0b013e31827e9289

[b5] Klaver Y. L. *et al.* Population-based survival of patients with peritoneal carcinomatosis from colorectal origin in the era of increasing use of palliative chemotherapy. Ann Surg Oncol 22, 2250–2256 (2011). 10.1093/annonc/mdq762 21345939

[b6] Chu E. An update on the current and emerging targeted agents in metastatic colorectal cancer. Clin colorectal cancer 11, 1–13 (2012). 21752724 10.1016/j.clcc.2011.05.005

[b7] Wang C. C. & Li J. An update on chemotherapy of colorectal liver metastases. World J gastroenterol 18, 25–33 (2012). 22228967 10.3748/wjg.v18.i1.25 PMC3251802

[b8] Franko J. *et al.* Treatment of colorectal peritoneal carcinomatosis with systemic chemotherapy: a pooled analysis of north central cancer treatment group phase III trials N9741 and N9841. J Clin Oncol 30, 263–267 (2012). 22162570 10.1200/JCO.2011.37.1039 PMC3269953

[b9] Klaver Y. L. *et al.* Outcomes of colorectal cancer patients with peritoneal carcinomatosis treated with chemotherapy with and without targeted therapy. Eur J Surg Oncol 38, 617–623 (2012). 22572106 10.1016/j.ejso.2012.03.008

[b10] Klaver Y. L. *et al.* Addition of biological therapies to palliative chemotherapy prolongs survival in patients with peritoneal carcinomatosis of colorectal origin. Am J Clin Oncol 36, 157–161 (2013). 22314003 10.1097/COC.0b013e3182438c55

[b11] van Gestel Y. R. *et al.* Metachronous peritoneal carcinomatosis after curative treatment of colorectal cancer. Eur J Surg Oncol 40, 963–969 (2014). 24183168 10.1016/j.ejso.2013.10.001

[b12] Kumar R. *et al.* Colorectal cancer survival: An analysis of patients with metastatic disease synchronous and metachronous with the primary tumor. Clin colorectal cancer 13, 87–93 (2014). 24373733 10.1016/j.clcc.2013.11.008

[b13] Slesser A. A. *et al.* The tumour biology of synchronous and metachronous colorectal liver metastases: a systematic review. Clin Exp Metastasis 30, 457–470 (2013). 23180209 10.1007/s10585-012-9551-8

[b14] Jayne D. G., Fook S., Loi C. & Seow-Choen F. Peritoneal carcinomatosis from colorectal cancer. Br J Surg 89, 1545–1550 (2002). 12445064 10.1046/j.1365-2168.2002.02274.x

[b15] Segelman J. *et al.* Incidence, prevalence and risk factors for peritoneal carcinomatosis from colorectal cancer. Br J Surg 99, 699–705 (2012). 22287157 10.1002/bjs.8679

[b16] van Gestel Y. R. *et al.* Metachronous peritoneal carcinomatosis after curative treatment of colorectal cancer. Eur J Surg Oncol 40, 963–969 (2014). 24183168 10.1016/j.ejso.2013.10.001

[b17] Cao C., Yan T. D., Black D. & Morris D. L. A systematic review and meta-analysis of cytoreductive surgery with perioperative intraperitoneal chemotherapy for peritoneal carcinomatosis of colorectal origin. Ann Surg Oncol 16, 2152–2165 (2009). 19434455 10.1245/s10434-009-0487-4

[b18] Elias D. *et al.* Complete cytoreductive surgery plus intraperitoneal chemohyperthermia with oxaliplatin for peritoneal carcinomatosis of colorectal origin. J Clin Oncol 27, 681–685 (2009). 19103728 10.1200/JCO.2008.19.7160

[b19] Verwaal V. J. Long-term results of cytoreduction and HIPEC followed by systemic chemotherapy. Cancer J 15, 212–215 (2009). 19556907 10.1097/PPO.0b013e3181a58d7c

[b20] Hurwitz H. I. *et al.* Bevacizumab in combination with fluorouracil and leucovorin: an active regimen for first-line metastatic colorectal cancer. J Clin Oncol 23, 3502–3508 (2005). 15908660 10.1200/JCO.2005.10.017

[b21] Kabbinavar F. F. *et al.* Addition of bevacizumab to bolus fluorouracil and leucovorin in first-line metastatic colorectal cancer: results of a randomized phase II trial. J Clin Oncol 23, 3697–3705 (2005). 15738537 10.1200/JCO.2005.05.112

[b22] Zani S. *et al.* Modest advances in survival for patients with colorectal-associated peritoneal carcinomatosis in the era of modern chemotherapy. J Surg Oncol 107, 307–311 (2013). 22811275 10.1002/jso.23222

[b23] Chua T. C. *et al.* Influence of modern systemic therapies as adjunct to cytoreduction and perioperative intraperitoneal chemotherapy for patients with colorectal peritoneal carcinomatosis: a multicenter study. Ann Surg Oncol 18, 1560–1567 (2011). 21203904 10.1245/s10434-010-1522-1

[b24] Folprecht G., Kohne C. H. & Lutz M. P. Systemic chemotherapy in patients with peritoneal carcinomatosis from colorectal cancer. Cancer Treat Res 134, 425–440 (2007). 17633071 10.1007/978-0-387-48993-3_28

